# An automatic segmentation and classification framework for anti-nuclear antibody images

**DOI:** 10.1186/1475-925X-12-S1-S5

**Published:** 2013-12-09

**Authors:** Chung-Chuan Cheng, Tsu-Yi Hsieh, Jin-Shiuh Taur, Yung-Fu Chen

**Affiliations:** 1 Department of Electrical Engineering, National Chung Hsing University, Taichung, Republic of China: Taiwan; 2 Division of Allergy, Immunology and Rheumatology, Taichung Veterans General Hospital, Taichung, Republic of China: Taiwan; 3 Department of Healthcare Administration, Central Taiwan University of Science and Technology, Taichung, Republic of China: Taiwan; 4 Institute of Biomedical Engineering and Material Science, Central Taiwan University of Science and Technology, Taichung, Republic of China: Taiwan; 5 Department of Health Services Administration, China Medical University, Taichung, Republic of China: Taiwan

## Abstract

Autoimmune disease is a disorder of immune system due to the over-reaction of lymphocytes against one's own body tissues. Anti-Nuclear Antibody (ANA) is an autoantibody produced by the immune system directed against the self body tissues or cells, which plays an important role in the diagnosis of autoimmune diseases. Indirect ImmunoFluorescence (IIF) method with HEp-2 cells provides the major screening method to detect ANA for the diagnosis of autoimmune diseases. Fluorescence patterns at present are usually examined laboriously by experienced physicians through manually inspecting the slides with the help of a microscope, which usually suffers from inter-observer variability that limits its reproducibility. Previous researches only provided simple segmentation methods and criterions for cell segmentation and recognition, but a fully automatic framework for the segmentation and recognition of HEp-2 cells had never been reported before. This study proposes a method based on the watershed algorithm to automatically detect the HEp-2 cells with different patterns. The experimental results show that the segmentation performance of the proposed method is satisfactory when evaluated with percent volume overlap (PVO: 89%). The classification performance using a SVM classifier designed based on the features calculated from the segmented cells achieves an average accuracy of 96.90%, which outperforms other methods presented in previous studies. The proposed method can be used to develop a computer-aided system to assist the physicians in the diagnosis of auto-immune diseases.

## Introduction

The immune system enables us to resist infections by counteracting invading organisms. Autoimmune disease is a disorder of immune system due to over-reaction of lymphocytes against one's own body tissues [1]. Common autoimmune diseases include Hashimoto's thyroiditis, rheumatoid arthritis, diabetes mellitus type 1, and lupus erythematosus. Anti-Nuclear Antibody (ANA) is an autoantibody produced by the immune system directed against the self body tissues or cells. The ANA test widely used to detect antibody in the blood plays an important role in the diagnosis of autoimmune diseases. When a particular antibody pattern has been detected, the patient may have the possibility of acquiring certain autoimmune diseases.

Indirect ImmunoFluorescence (IIF) technique applied on HEp-2 cell substrates provides the major screening method to detect ANA patterns in the diagnosis of autoimmune diseases. It produces the ANA images with distinct fluorescence intensities and staining patterns through IIF slides. Currently, the ANA patterns are inspected by experienced physicians to identify abnormal cell patterns, which is a laborious task and may cause harm to physicians' eyes. It is not easy to train a qualified physician in a short term. Furthermore, manual inspection suffers from the difficulties, such as intra- and inter-observer variability, that limit the reproducibility of IIF readings [2-5].

Although previous studies have proposed several methods for automatic segmentation of ANA cells [6,7] and criteria for recognition of cell patterns [3,6,8-10], a fully automatic segmentation and recognition framework has never been developed so far. In this study, we propose a framework based on the watershed approaches to automatically segment the HEp-2 cells. It is a crucial preprocessing step for a computer aided system to classify the cell patterns to provide information to assist physicians in disease diagnosis and treatment.

Since the cytoplasm of HEp-2 cells is invisible in the IIF images, in what follows, the term "cell" means cell nucleus, "foreground" indicates the cell region, and "background" denotes the rest of the image. The rest of this paper is organized as follows. Section "Related Works" reviews the techniques used for ANA image segmentation and cell recognition in previous studies. Section "Segmentation of ANA Cells" describes the methods proposed in this study for the segmentation of ANA cells. Classification of ANA cell patterns is demonstrated in section "Cell Classification of ANA Images". Finally, discussions, conclusions, and future works are made in sections "Discussion" and "Conclusion and Future Work".

## Related works

In this section, the methods proposed in previous investigations for the segmentation and classification of ANA cell images are presented.

### ANA image segmentation

Perner *et al*. [6] used image processing techniques, including image transformation, histogram equalization, Otsu thresholding [11], and morphological operation, to obtain a binary mask for segmenting the cells from the ANA images. By modifying the methods, Huang *et al*. [7] presented two adaptive automatic segmentation frameworks to precisely extract the ANA cells. In their studies, the first framework classified an image into two categories, i.e., sparse and mass cell regions, based on the number of connected regions. Depending on the category of the images, different color spaces and processing techniques were adopted for cell segmentation. Morphological operations were also used to obtain smooth segmentation results. It was demonstrated to be able to deal with the segmentation of different patterns of IIF images. On the other hand, in the second framework, watershed segmentation [12] was applied on the green channel of the RGB images, followed by region merging and elimination to obtain the cell boundaries. If the number of regions in the obtained image was larger than a pre-defined threshold, the framework converted the original image into CMY color space and performed marker-controlled watershed segmentation [13] on the cyan color component. It was reported that the segmentation performance achieved an overall sensitivity of 94.7%.

Creemers *et al*. [14] proposed a unsupervised segmentation algorithm, based on iterative global Otsu thresholding and morphological opening operation, to support IIF testing. It was reported to have the capability to split connected regions into individual regions with an average accuracy of 89.57%.

### ANA cell recognition

Perner [8] presented the first study on fluorescent image analysis, feature extraction and classification. Then, an automatic cell recognition approach based on a variety of features, including size, color density, and number of cells, extracted from the segmented images was proposed [6]. For the cells with identical color density, features including standard deviation, mean shape factor, mean of perimeter, and standard deviation of perimeter, were further extracted. Data mining techniques, including Boolean model and decision tree induction, were then used to label the cell regions. Finally, human experts tagged each labeled region with a semantic label. Based on the aforementioned methods, Sack *et al*. [3] presented a system to automatic classify HEp-2 fluorescent patterns with a classification accuracy greater than 83%.

According to the fluorescence intensity, Soda and Iannello [9] classified the ANA images into a variety of patterns. They further proposed a framework consisting of hybrid rule-based multi-expert systems for the classification of ANA patterns with an overall error rate of 2.7-5.8% [15]. The framework extracted the features including the first, second, and fourth moments of the gray-level co-occurrence matrix, Zernike moments, as well as the coefficients of discrete cosine transform (DCT) and discrete wavelet transform (DWT). Based on the efforts of previous researches, Rigon *et al*. [16] proposed a comprehensive system based on two approaches, in which the first approach discriminated the positive cells from the negative and weakly positive cells based on the features of fluorescence intensity, whereas the second one recognized the staining pattern of the positive cells. The performance of positive/negative recognition ranges from 87% to more than 94%, whereas the staining pattern classification accuracy of the main classes, i.e. homogeneous cells, peripheral nuclear cells, speckled cells, nucleolar cells, and artefacts, ranges from 71% to 74%.

Elbischger *et al*. [17] developed an iterative thresholding algorithm for processing HEp-2 cells and a cell classifier for detecting auto-immune diseases. Features including area to perimeter ratio, variance, 30th and 60th normalized percentiles, percentile range, dent number, auto-covariance percentage, and roundness, were extracted from the segmented cells and used for cell classification. The system was reported to be capable of distinguishing 5 different patterns with an overall accuracy of 93% based on the dataset consisting of 982 ROIs extracted from 38 images.

Recently, Huang *et al*. [18] employed the self-organizing map (SOM) to identify the fluorescence patterns of HEp-2 cells. Fourteen features, including the perimeter, area, and histogram uniformity of the cell; area and average intensity of the inside and perimeter areas of the cell; higher and lower intensity ratios of the inside area, perimeter area, and whole area of the cell; and standard deviation of the inside area of the cell, were used for designing the classifier with an average accuracy of 92.4%. In [19], the EUROPattern designed based on k-nearest neighbor algorithm, was compared with the conventional visual IIF evaluation with the sensitivity and specificity achieving 100% and 97.5%, respectively. In addition, it was shown that 94.0% of all the main antibody patterns, including the positive patterns, i.e., homogenous, speckled, nucleolar, centromere, nuclear dotted, and cytoplasmic patterns, as well as the negative patterns, could be correctly recognized.

## Segmentation of ANA cells

**Figure 1:**
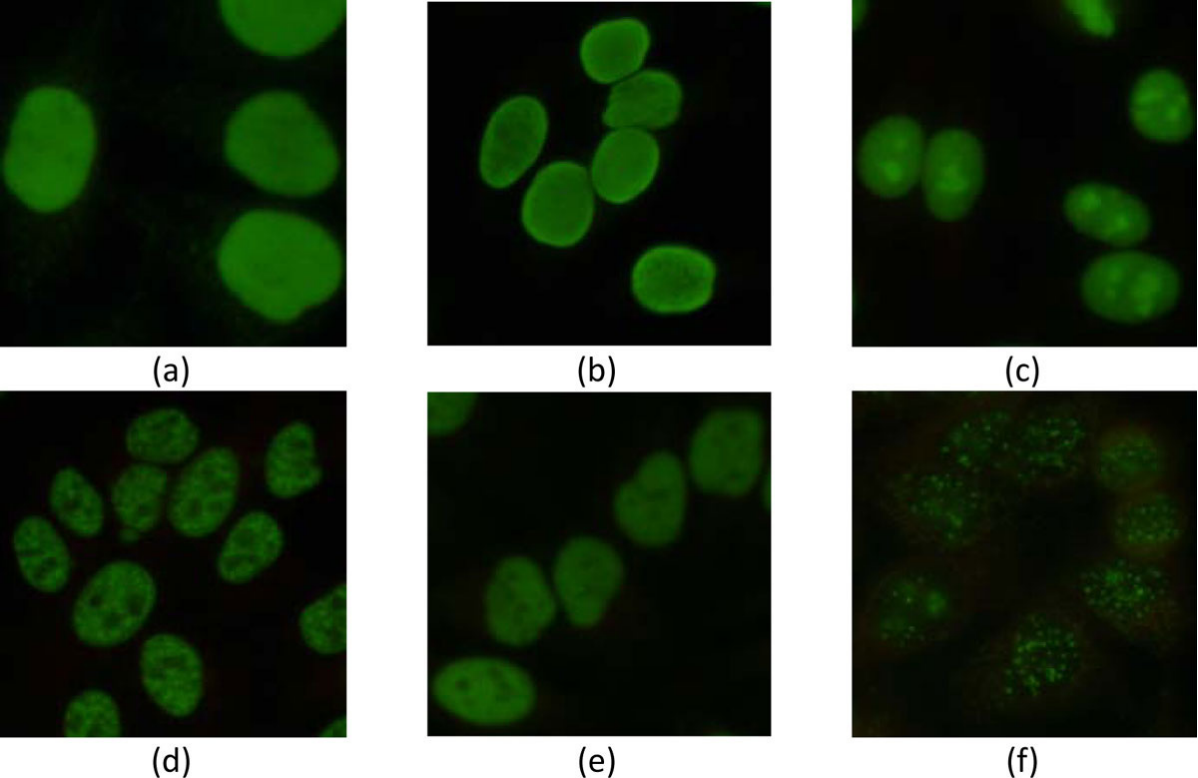
**Example of ANA images**. Images classified into (a) diffused, (b) peripheral, (c) nucleolar, (d) coarse-speckled, (e) fine-speckled, and (f) discrete-speckled patterns.

**Figure 2:**
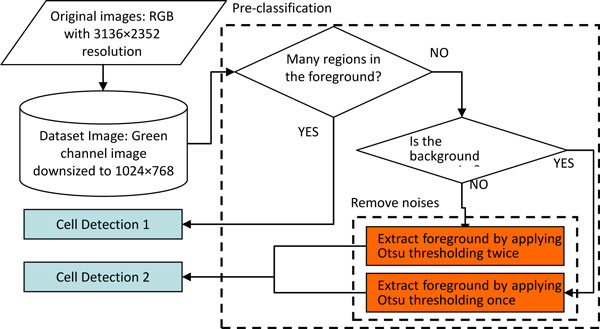
**Procedure of the proposed method**.

As recommended by Center for Disease Control (CDC) [20,21], in this study, the IIF slides were prepared at 1:80 serum dilution, and the ANA images were acquired by a digital camera mounted on a fluorescence microscope at a 40-fold zoom. The images were stored with the format of 24-bit RGB color depth and a resolution of 3136×2352 pixels. As shown in Figure 1, the ANA cells are classified into six categories: diffused, peripheral, nucleolar, coarse-speckled, fine-speckled, and discrete-speckled patterns. A dataset, consisting of 196 images classified into 37 diffused, 29 peripheral, 5 nucleolar, 94 coarse-speckled, 1 fine-speckled, and 30 discrete-speckled images by an expert (Dr. Hsieh), was used for the experiments. The procedure of the proposed method is illustrated in Figure 2.

**Figure 3:**
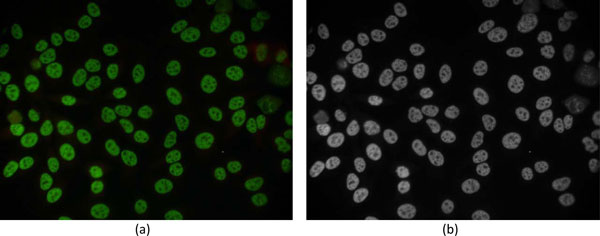
**Green-channel image**. Example of (a) an original ANA image and (b) its green-channel image.

Since the original images are stained by green dye, the proposed method extracts only the green channel from the original RGB ANA images for processing. In order to reduce the computation time, the images are downsized from 3136×2352 to 1024×768 pixels. It was found that images at this resolution can still provide enough information for the segmentation and classification of cell patterns. Figure 3 shows an example of ANA image and its corresponding green-channel. As described in the following 3 subsections, the proposed segmentation method divided into 3 procedures, i.e. pre-classification, cell detection 1, and cell detection 2, is presented. The parameters used for these 3 procedures are described in subsection "Parameters for cell segmentation". Finally, the segmentation results are demonstrated in subsection "Segmentation results".

### Pre-classification

Automatic segmentation of ANA images cannot be handled in a unified way because the characteristics of the images in different categories are quite dissimilar. For example, the discrete speckled cells look like irregular broken blobs and are significantly different from the cells of other 5 categories that appear as elliptic blobs but still having diverse appearances (cf. Figure 1). Thus, the images are pre-classified according to their differences in image patterns before conducting cell segmentation. In the pre-classification stage, the images are divided into two groups. Images with larger grey-level variance or more regions contained in the foreground are assigned to the first group, and the rest of the images are assigned to the second group. The images in these two groups are segmented using different methods as detailed in subsections "**Cell detection 1**" and "**Cell detection 2**". The procedure of pre-classification is summarized as follows:

1) First, Otsu thresholding algorithm is used to roughly separate the foreground regions from the background.

2) The *closing *morphological operation is employed to fill the holes and to eliminate small regions in the foreground.

3) If the number of foreground regions in an image is larger than the threshold, ***th_num***, or its foreground regions contain staining noises with variance higher than the threshold, ***th_fg_var***, it is segmented using "Cell detection 1"; otherwise "Cell detection 2" is adopted. In this study, the thresholds ***th_num ***and ***th_fg_var ***are set to 200 and 1000, respectively.

4) For images segmented with "Cell detection 2", the staining noise in the background regions are removed according to their noise level, as defined in the following equation:

$$\overset{128}{\underset{i=0}{\sum }}\left[p\left(i\right)>0\right],$$

where *i *indicates the gray level of the image, and *p*(*i*) denotes the frequency of gray level *i *in the image. The threshold of noise level, ***th_noise***, is set to 10.

### Cell detection 1

**Figure 4:**
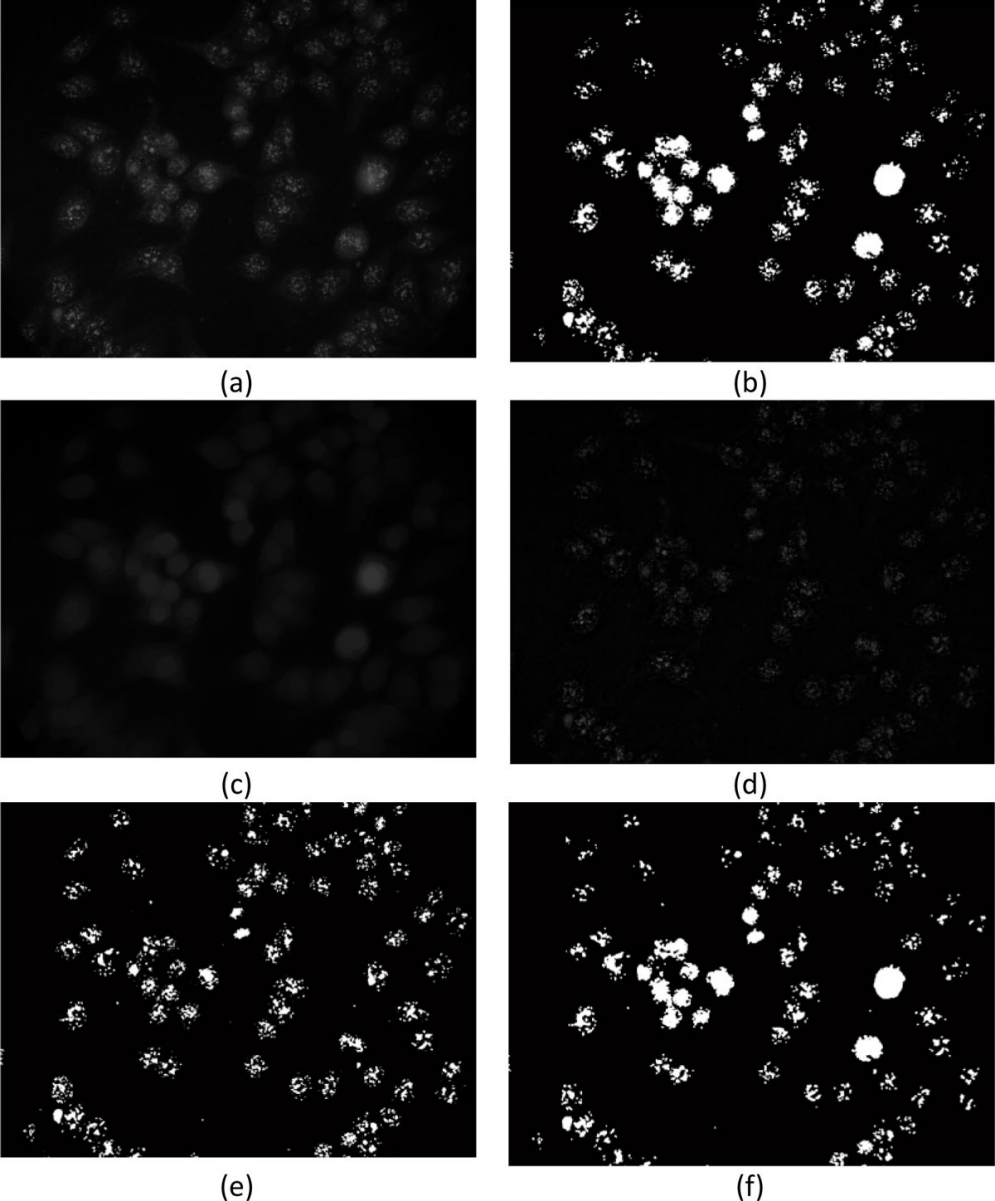
**Extraction of marker images**. (a) The original, (b) initial marker, and (c) smooth images. (d) Difference image obtained from the original image and the smooth image. (e) Image after performing Otsu thresholding on difference image and (f) the marker image after applying opening morphological operation.

The approach is designed for cell detection of the images containing more foreground regions or the gray level in the foreground regions presenting great variance. It consists of two stages: image segmentation and cell extraction. Cells are extracted according to the cell contours obtained from the general watershed segmentation [12] and marker-controlled watershed segmentation [13]. As described below, the procedure of this approach is divided into four steps. Figure 4 illustrates the results obtained from individual steps.

**Figure 5:**
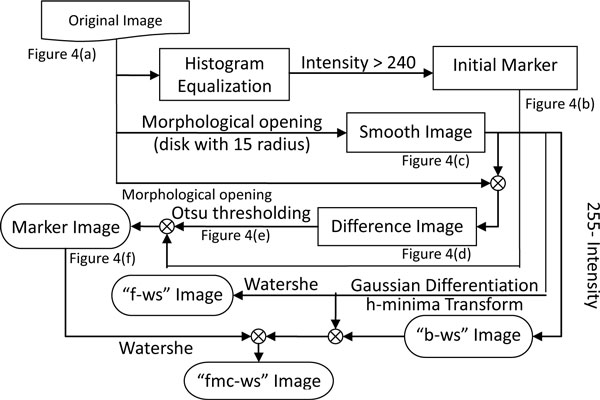
**Flowchart of marker extraction**.

1) The histogram equalization is applied to the original image in Figure 4(a), and then the pixels with gray level greater than 240 are considered as the initial markers, as presented in Figure 4(b).

2) As demonstrated in Figure 4(c), the original image is smoothed by the morphological opening operation using a disk-shaped structuring element with a radius of 15.

3) The Difference between the original image and the smoothed image is obtained, Figure 4(d), and is converted into a binary image, Figure 4(e), by applying the Otsu thresholding method.

4) The initial markers are superimposed on the thresholded image shown in Figure 4(e), followed by applying the same opening morphological operation mentioned in the 2^nd ^step to obtain the marker image, Figure 4(f), used for marker-controlled watershed segmentation. The flowchart of marker extraction is depicted in Figure 5.

As described in Steps 5-7, 3 types of watershed images are obtained based on the original image, smooth image, and marker image, and are used for cell segmentation.

**Figure 6:**
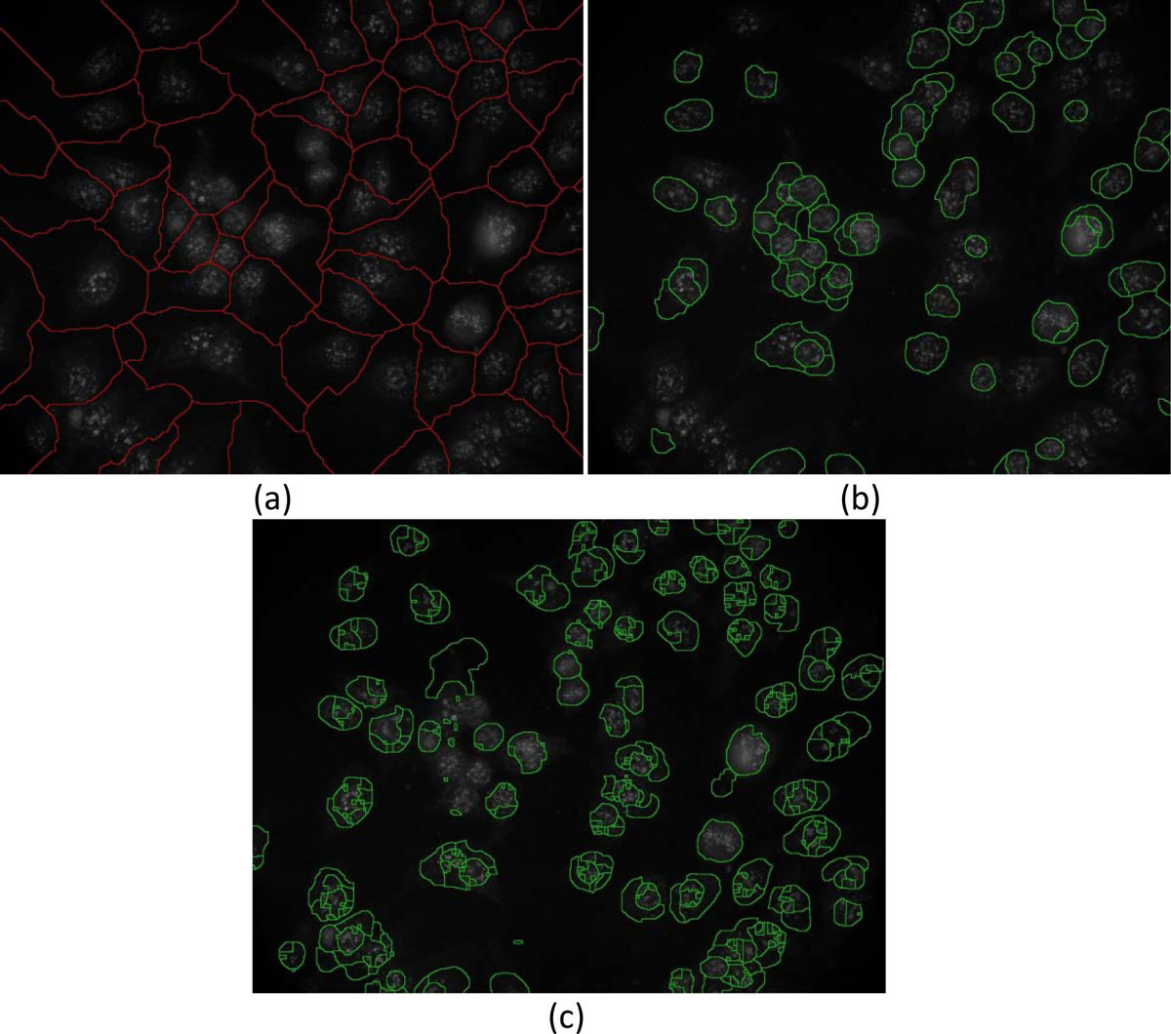
**Examples of three types of watershed images**. (a) Background-watershed (b-ws) image, (b) foreground-watershed (f-ws) image, and (c) foreground marker-controlled-watershed (fmc-ws) image superimposed on the original image.

5) The original image shown in Figure 4(a) is complemented by subtracting each pixel value from 255 for conducting watershed segmentation. Figure 6(a) shows the background watershed image (b-ws) superimposed on the original image.

6) Furthermore, Gaussian differentiation parameter with *σ*=2 and thresholding parameter of *h*-minima suppression with ***th_h1 ***= 0.12 [22] are applied on the smoothed image for conducting watershed segmentation to obtain the foreground watershed segmentation image (f-ws). Figure 6(b) presents the "f-ws" image superimposed on the original image.

7) Similar to the foreground watershed segmentation, the smoothed image is first filtered by the Gaussian differentiation and minima suppressed by *h*-minima transform, which is then superimposed by the marker image and the "b-ws" image to obtain the foreground marker-controlled watershed (fmc-ws) image used for marker-controlled watershed segmentation. Figure 6(c) shows the foreground marker-controlled watershed (fmc-ws) image.

These 3 types of watershed images were further used for cell segmentation. As demonstrated in Figure 6, it can be observed that the "b-ws" image is effective in splitting cells that are close to each other. The blobs in "fmc-ws" are mostly over-segmented with unsmooth contours, resulting in a failure to effectively delineate the cell contours. On the other hand, the "f-ws" image is unable to detect some of the cell regions. Consequently, in the cell extraction stage of "Cell detection 1", the three types of watershed images and the marker image are combined to precisely extract cell boundaries.

**Figure 7:**
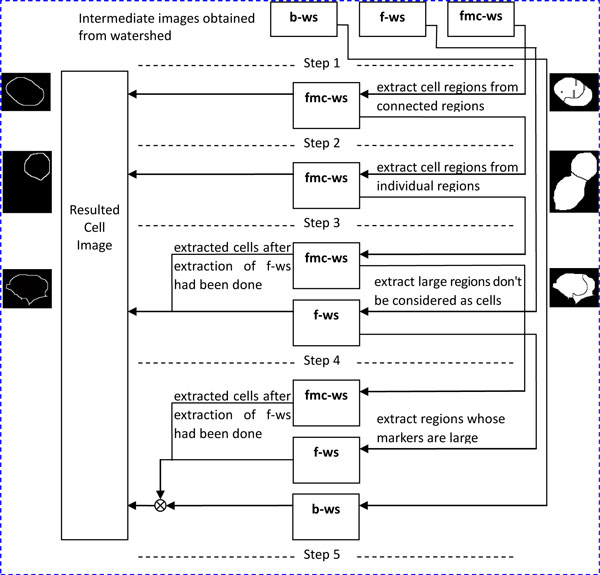
**Procedure of cell extraction in "Cell detection 1"**.

**Figure 8:**
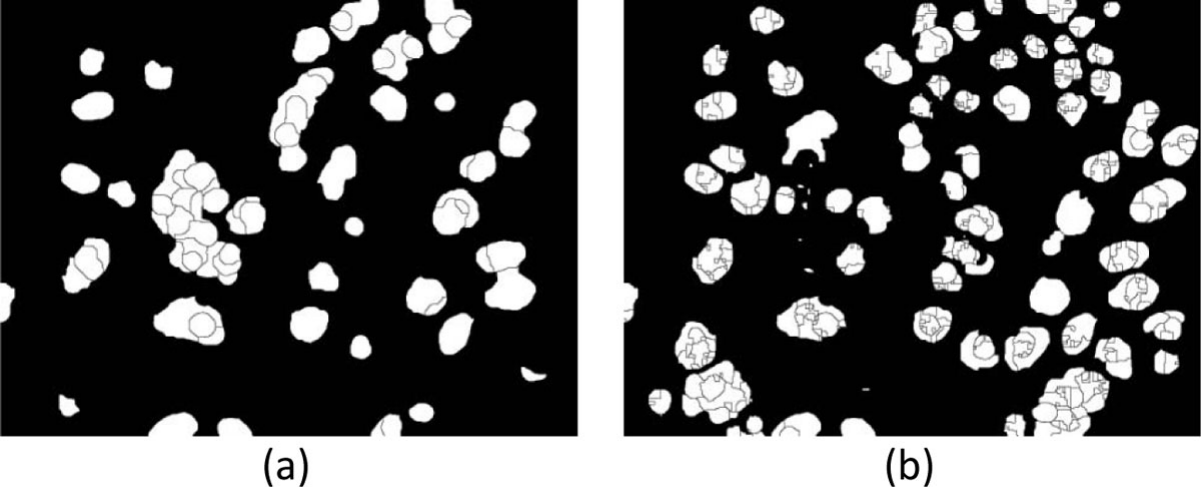
**Mask images of cell regions with background removal**. (a) "f-ws" image and (b) "fmc-ws" image.

**Figure 9:**
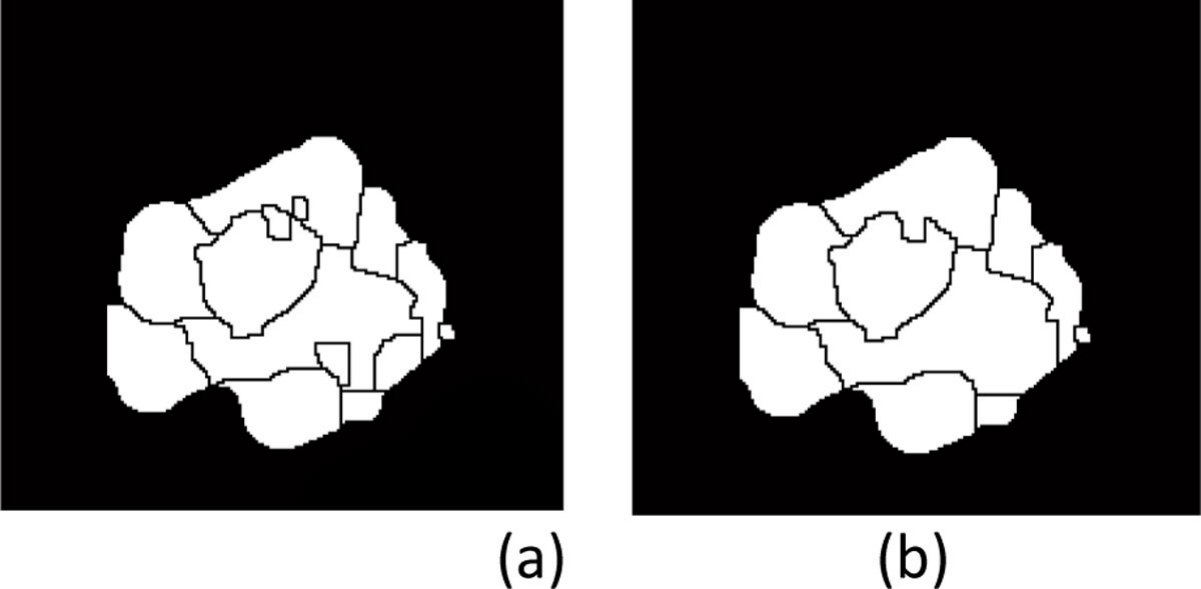
**Merging the small inner regions of a region not justified as a cell**. (a) Inner regions which are not justified as cells and (b) larger inner regions obtained by merging smaller inner regions with morphological closing operation.

**Figure 10:**
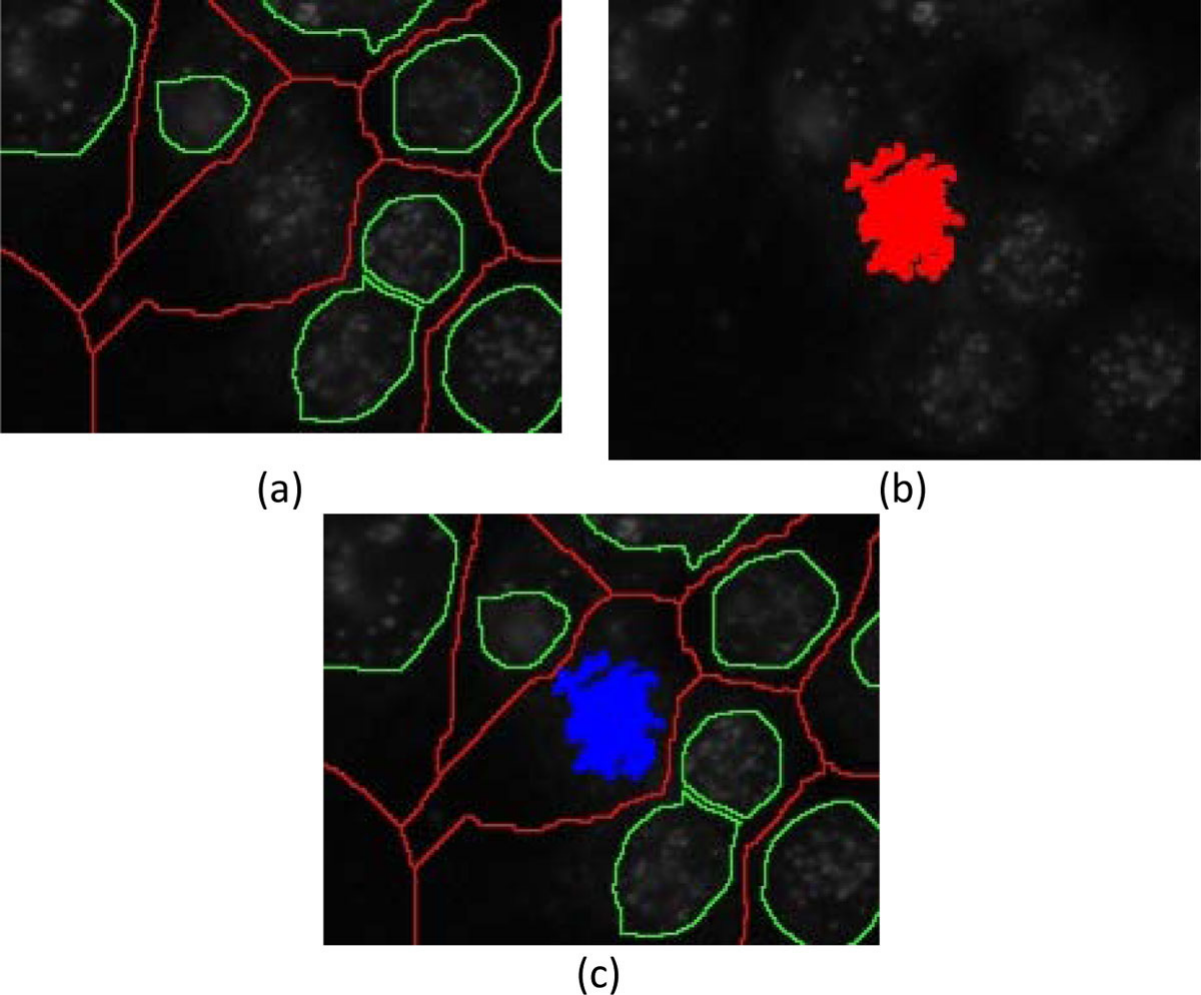
**Extracting cells for regions with large markers**. Illustration of (a) 8 detected cells (green) and 1 undetected cell, (b) the marker corresponding to the b-ws region containing the undetected cell, and (c) superimposition of images shown in (a) and (b).

As illustrated in Figure 7, the strategies for cell extraction using the watershed images are described in the following steps:

1) The three watershed images, i.e., "b-ws", "f-ws" and "fmc-ws", are all binary images. The cell contours in "f-ws" and "fmc-ws" images are labeled as ZERO, otherwise ONE, followed by the removal of background regions to obtain the watershed mask images shown in Figure 8(a) and 8(b), respectively.

2) The cell regions are extracted from the "fmc-ws" image according to the perimeters of the connected regions, since it can potentially detect more cell regions than "f-ws". The regions whose areas larger than the threshold ***th_area ***are justified by "ellipse test" and considered as the cells after having passed the test.

3) For the regions with areas smaller than the threshold ***th_area***, closing morphological operation is conducted to merge smaller regions. The merged regions are then justified by ellipse test for cell extraction. As demonstrated in Figure 9, the small inner regions of the remains are merged to larger regions.

4) For the regions, which are not deemed as ellipses from the remains of the "fmc-ws" at Step 3 and the "f-ws", having areas higher than ***th_area ***and containing markers in the corresponding locations, they should be treated as the candidate cells. Due to the fact that the blobs of "f-ws" are more similar to real cells than "fmc-ws", "f-ws" is used to perform cell extractions before applying "fmc-ws" here.

5) Most of the cells in "f-ws" and "fmc-ws" should have been extracted at the previous 4 steps, but some regions may not be detected because their markers are large enough to cover the edges of the regions. Figure 10(a) demonstrates the cells detected at Steps 1-4. However, as shown in Figure 10(b), watershed segmentation may fail when detecting the cells whose corresponding markers are large enough to cover the whole candidate cell. Hence, if the markers existed in the marker-image are larger than the threshold ***th*_*area*2**, the watershed segmentation (with a parameter of *h*-minima transform ***th*_*h*2**) is performed in the corresponding region of the smooth image. Here, only the corresponding region of "b-ws", as shown in Figure 10 (c), is considered for extracting the cells.

**Figure 11:**
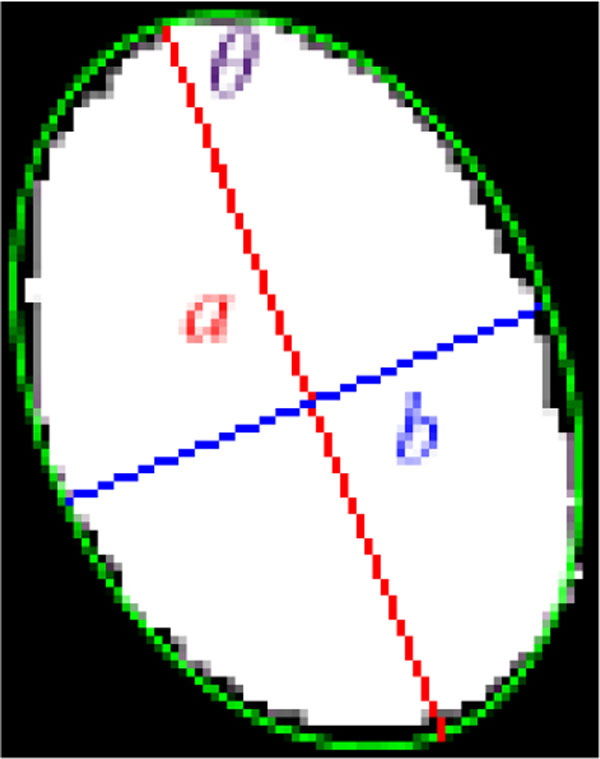
**A region *r_i _*and its fitted ellipse *r_i_^I^*(green)**.

Due to the fact that the real HEp-2 cells usually appear as ellipses, the cells can be justified by "ellipse test". It is used to justify whether a region contains a cell or not. Given a region *r_i_*, the error between *r_i _*and a real ellipse *r_i_^I ^*is defined as:

$$e_{i}=\frac{\left|{r_{i}}^{\prime }\right|}{\left|r_{i}\right|},\text{with }{r_{i}}^{\prime }=r_{i}\;\text{XOR }r_{i}^{I}\;\text{and }r_{i}^{I}=\text{Ellipse}\left(a,b,\theta \right)$$

in which |*r_i_*| denotes the number of pixels in *r_i _*and *r_i_^I ^*is the estimated ideal ellipse for *r_i_*, comprising three parameters: major-length (*a*), minor-length (*b*), and orientation (*θ*). The lengths of major axis and minor axis are both computed according to the centroid of *r_i_*. If the error function of a region is equal to zero, the region is deemed as an ideal ellipse. Figure 11 depicts a region and its estimated ideal ellipse. If the error of a region is lower than the threshold ***th_error***, it is marked as a cell; otherwise, it may be treated as one of following cases: not a cell, an incomplete cell, or a connected region.

### Cell detection 2

**Figure 12:**
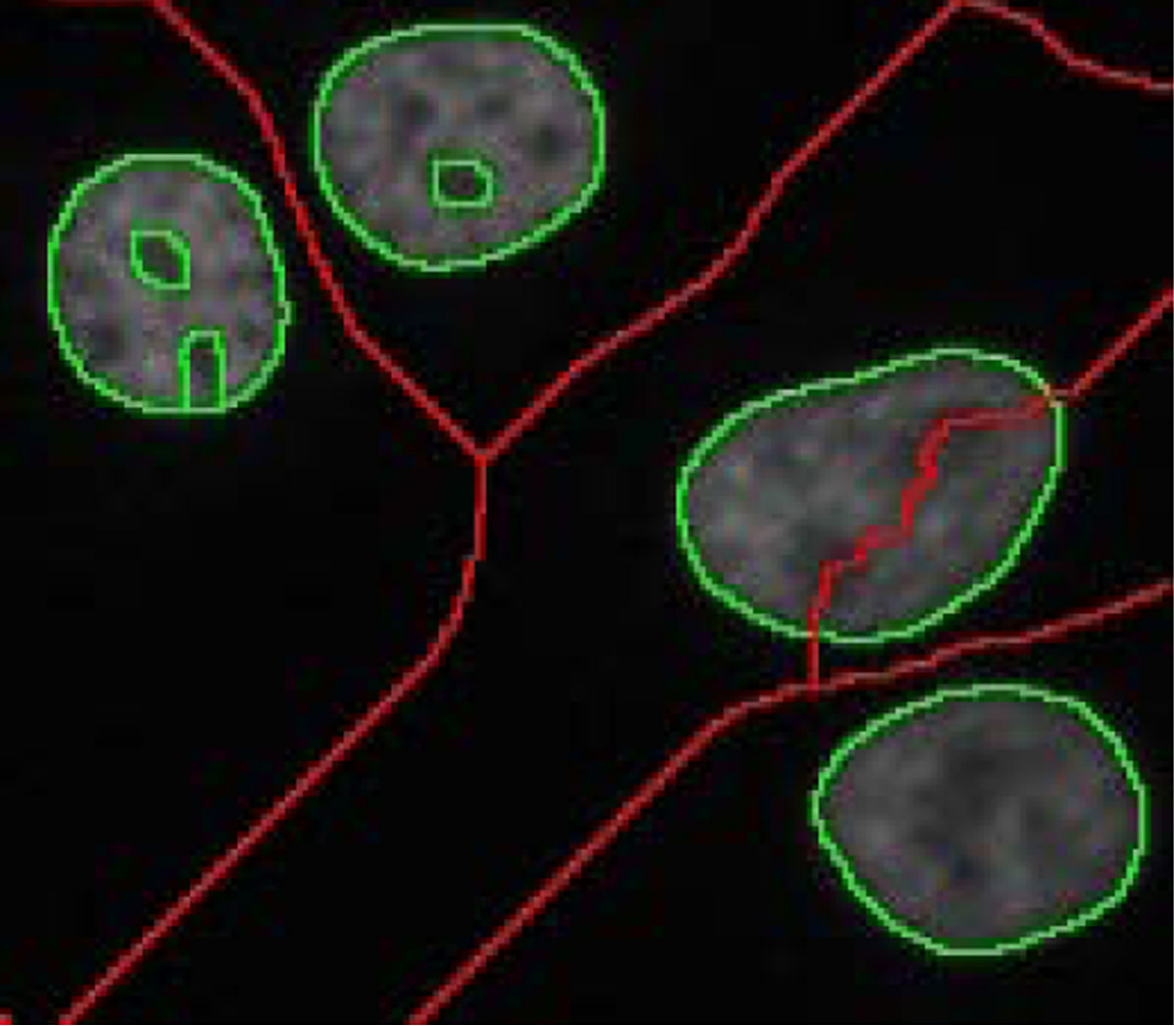
**An example of cell segmentation using "Cell Detection 2"**.

**Figure 13:**
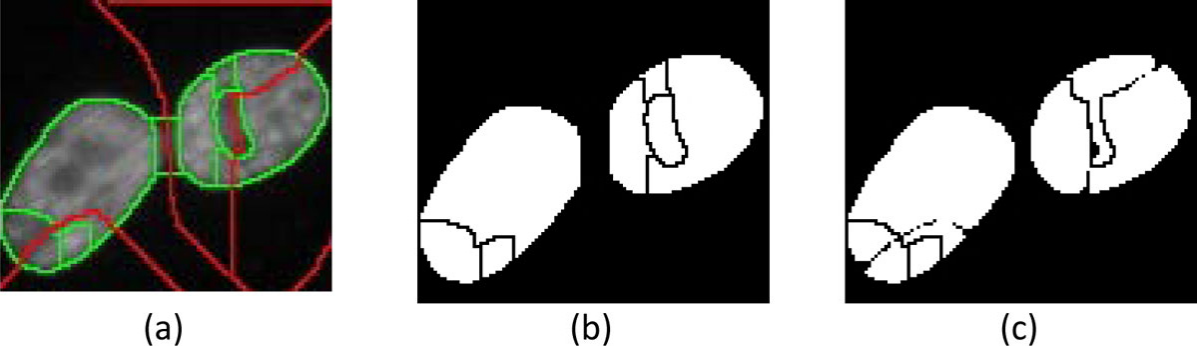
**Splitting of a fake sub-region**. (a) A fake sub-region with a line in "b-ws" crossing it is split to (b) two separated candidate cell regions. (c) Subsequent watershed segmentation is performed on the candidate cell regions on "f-ws".

**Figure 14:**
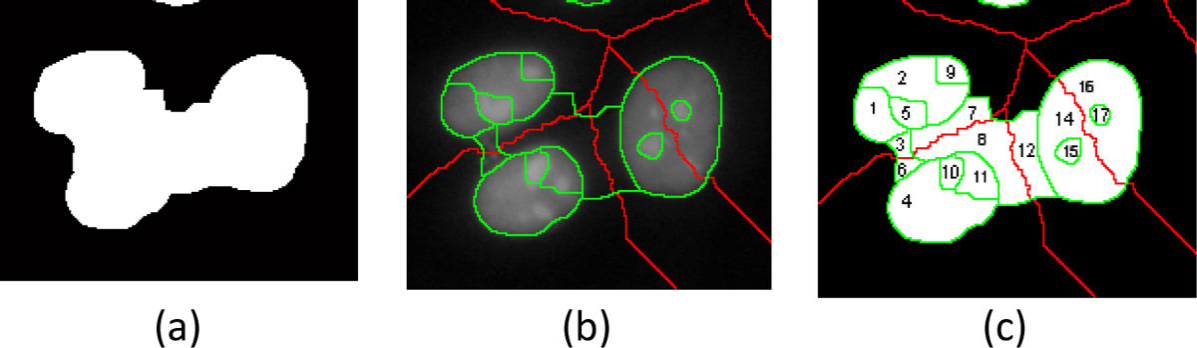
**Example of splitting a connected region**. (a) A connected region is split into 3 cell regions by (b) superimposing "b-ws" image on "f-ws" image and then (c) determining combined sub-regions and discarded sub-regions.

The approach is applied to the images containing less foreground regions (***th_num***<200) and less staining noise (***th_fg_var***<1000) detected in the foreground. As shown in Figure 12, the procedure is very similar to that in "Cell detection 1", except that the image segmentation uses only the "b-ws" (red) and "f-ws" (green) watershed images without considering the "fmc-ws" image. The procedure is described as follows:

1) Remove the background regions of the "f-ws" image.

2) Extract cell regions from "f-ws".

3) Because of the characteristics of watershed segmentation, the adjacent regions form connected regions. The regions which are not extracted in step 2 may be fake connected regions, which can be split by using the information embedded in the "b-ws" image. As illustrated in Figure 13(a), a sub-region, which connects two watershed regions, with a line in the "b-ws" image crossing it will be eliminated, resulting in the separation of two cell blobs, Figure 13(b). Subsequently, watershed segmentation (with a designated parameter of h-minima transform, ***th_h1***) is further performed on the individual cell regions appeared on "f-ws", Figure 13 (c). The sub-regions in the refined cell regions are merged and justified by "ellipse test" afterward.

4) For connected cell regions which can't be split at step 3, all possible combinations of sub-regions will be tested to obtain combinations of sub-regions which are similar to ellipses. Once the best combination has been obtained, the cell regions can be well-separated from the background. Figure 14 illustrates the procedure in splitting the region containing three candidate cell regions. A connected region *r_i _*consisting of *N_i _*sub-regions can be indicated as:

$$r_{i}=\left\{r_{i,1},r_{i,2},\cdots r_{i,j},\cdots r_{i,N_{i}}\right\}$$

The error function of the *k*_th _combination of sub-regions, *comb_k_*, can be calculated according to:

$$e_{k}=\frac{\left|comb_{k}^{{}^{\prime }}\right|}{\left|comb_{k}\right|},$$

where $comb_{k}^{\prime }=comb_{k}\text{ XOR }comb_{k}^{I}$ with $comb_{k}^{I}$ denoting the estimated ideal ellipse of *comb*_*k*_. If the combination with smallest error, $k^{\prime }=\arg\underset{k}{\min}e_{k}$, has been found, the connected region can be split to isolated regions *r_i_*' accordingly. As shown in Figure 14(c), "b-ws" image is superimposed on the "f-ws" image to form 17 sub-regions. The combinations with smallest errors include {*r*_*i,1*_, *r*_*i,2*_, *r*_*i,5*_, *r*_*i,9*_}, {*r*_*i,4*_, *r*_*i,10*_, *r*_*i,11*_}, {*r*_*i,14*_, *r*_*i,15*_, *r*_*i,16*_, *r*_*i,17*_}, and {*r*_*i,3*_, *r*_*i,6*_, *r*_*i,7*_, *r*_*i,8*_, *r*_*i,12*_}. After the ellipse tests, the sub-region combinations {1, 2, 5, 9}, {4, 10, 11}, and {14, 15, 16, 17} are merged into 3 cell regions, while the combination {3, 6, 7, 8, 12} is discarded. Once a connected region has been split, the new regions are modified by performing watershed segmentation (with a designated parameter of *h*-minima transform, ***th***_***h2***) in their locations corresponding to the locations in the "b-ws" image. The sub-regions {3, 6, 7, 8, 12} are discarded because their intensities and textures are very similar to the background when the local watershed segmentation has been applied.

5) Due to the fact that the foreground of dataset images may contain inhomogeneous gray levels, some regions can not be detected because they are darker than other regions, even though they can be discriminated by human eyes. In order to detect these regions, global Otsu thresholding is again performed on the remaining image after cell extraction. Detected regions with areas greater than ***th_area ***are considered as cells.

### Parameters for cell segmentation

The parameters used for different stages of cell segmentation are listed in Table 1. The parameters ***th_h1 ***and ***th_h2 ***are crucial in effectively suppressing noises and local irregularities in the gradient images. Furthermore, the segmentation results are very sensitive to the parameters, even they are only changed slightly; hence they are set case by case for obtaining complete blobs and avoiding over-segmentation. If the values are too small, the blob will be over-segmented and need more time to find *r_i_*', which is the combination of sub-regions with the smallest error. In contrast, larger values may cause the watershed to reach a boundary outside the blob and cannot converge at the real boundaries. The procedures of setting the parameters ***th_h1 ***and ***th_h2 ***are based on the greedy algorithm.

**Table 1:** Parameters designated for different stages of cell segmentation.

Pre-classification		Cell Detection 1		Cell Detection 2	
**Parameter**	**Value**	**Parameter**	**Value**	**Parameter**	**Value**

***th_num***	200	***th_h1***	0.12	***th_h1***	0.12

***th_noise***	10	***th_h2***	0.28	***th_h2***	0.28

***th_fg_var***	1000	***th_area***	400	***th_area***	400

		***th_error***	0.095	***th_error***	0.095

		***th_area2***	32		

**Figure 15:**
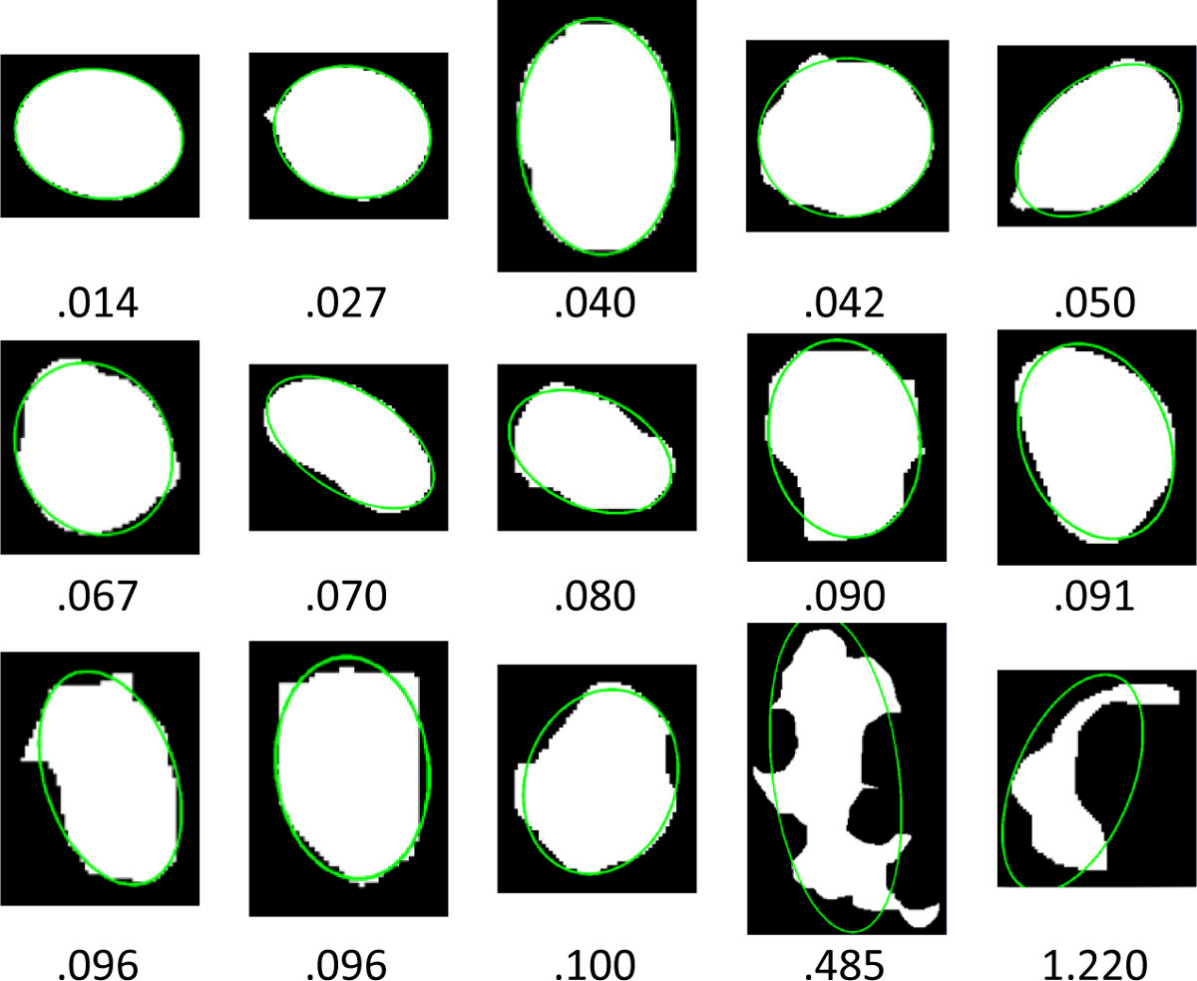
**Comparisons of *th_error *values among regions with different shapes**.

The parameters, ***th_area ***and ***th_error***, are used as the criteria for judging whether a blob is a cell or not. Considering an ANA image with a size of 1024×768 pixels, the minimum cell size is set to 400 pixels, i.e. ***th_area ***= 400, according to the physician's opinion. Figure 15 compares the errors among regions with different shapes. Note that a perfect ellipse has a zero error. Since HEp-2 cells may be squeezed, superimposed, demolished, or deviated from a perfect ellipse, the value of ***th_error ***(0.095) is determined by greedy algorithm with a grid size of 0.005 to select the optimal threshold with best detecting accuracy according to the 3830 ground-truth cell images extracted from 196 images in the dataset. On the other hand, the parameter ***th*_*area*2 **is used to find the markers located in non-recognized cell regions. In the cases of nucleolar and discrete-speckled patterns, the markers could be too small to be used for cell detection. Hence, its value is assigned as ***th*_*area*2 **= 32 for mild restriction.

### Segmentation results

**Figure 16:**
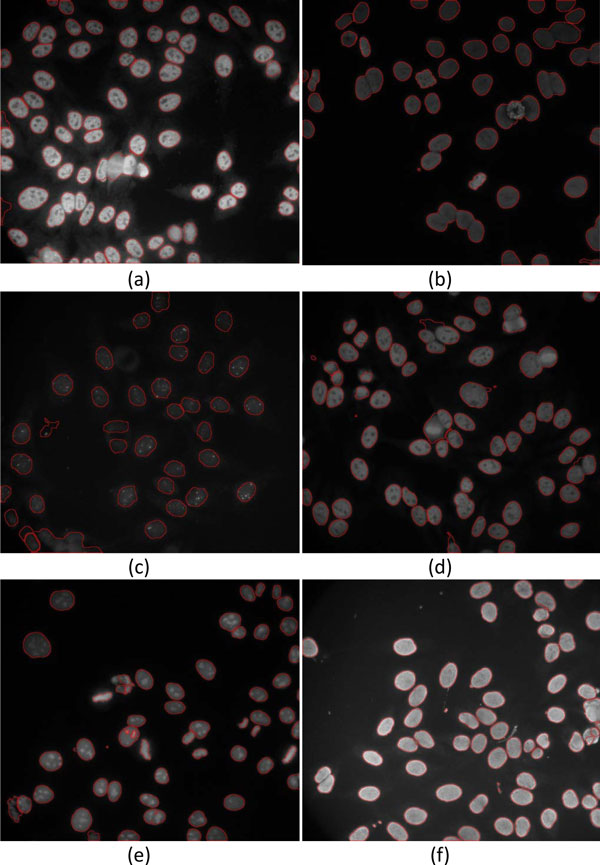
**Examples of segmentation results of ANA images**. Segmented cells of images with (a) coarse-speckled, (b) diffused, (c) discrete-speckled, (d) fine-speckled, (e) nucleolar, and (f) peripheral patterns, respectively.

**Figure 17:**
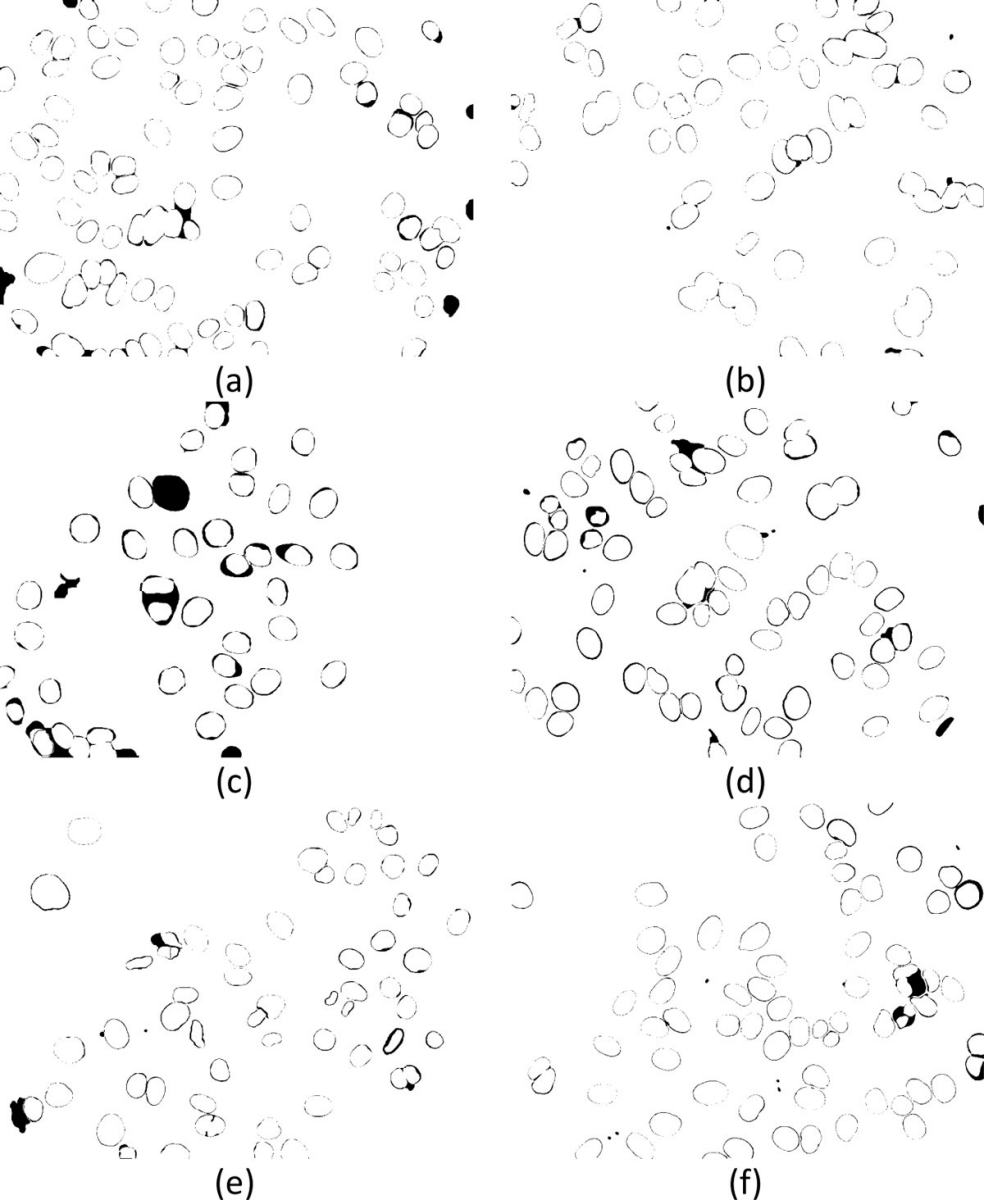
**Comparisons of segmentation results between proposed method and ground-truth**. Segmented results of images with (a) coarse-speckled, (b) diffused, (c) discrete-speckled, (d) fine-speckled, (e) nucleolar, and (f) peripheral patterns overlapped on ground-truth images.

Figure 16 demonstrates the segmentation results of ANA images with 6 different patterns. As shown in this figure, the proposed method performs well on almost all the images with different cell patterns; however, the performance on images of diffuse and discrete-speckled patterns is less satisfactory because the cells of diffused pattern contain more closely connected regions than the other types of cells, whereas the cells of discrete-speckled pattern appear to have less obvious boundaries. Figure 17 compares the segmentation results using the proposed method with examples of the ground-truth images. The ground-truth images were delineated by the technicians trained by one of the authors, Dr. Hsieh. Performance of the segmentation results was evaluated with percent volume overlap (PVO) and percent volume difference (PVD) that had been used widely in previous works [23-26].

Given two contours, denoted by *C_s _*and *C_g_*, obtained from the proposed method and the ground truth, respectively, of the segmented image, PVO and PVD can be calculated from the following formula:

$$\begin{array}{c} PVO\left(C_{s},C_{g}\right)=\frac{V\left(C_{s}\cap C_{g}\right)}{\left[V\left(C_{s}\right)+V\left(C_{g}\right)\right]/2}\times 100\%,\;\text{and}\\ PVD\left(C_{s},C_{g}\right)=\frac{\left|V\left(C_{s}\right)-V\left(C_{g}\right)\right|}{\left[V\left(C_{s}\right)+V\left(C_{g}\right)\right]/2}\times 100\%, \end{array}$$

where *V*(*C*) indicates the volume of a contour. Table 2 presents the average performance of the segmentation results evaluated based on PVO and PVD. The results show that the proposed method can detect cells accurately in most image cases with the PVO greater than 89% and the PVD less than 22%. Even for the most difficult cases appear in cells with discrete-speckled pattern, PVO can still achieve a value over 75%. As a matter of fact, it is not necessary to segment HEp-2 cells with great accuracy. However, the segmentation results must be good enough to extract features to support accurate cell classification, as described in next section.

**Table 2:** Comparisons of cell segmentation performance

Pattern	Cell Number	PVO (%)	PVD (%)
Coarse speckled	260	92.35	15.31

Diffuse	157	87.89	24.23

Discrete speckled	195	78.03	43.95

Fine speckled	67	91.27	17.46

Nucleolar	175	91.94	16.12

Peripheral	153	93.47	13.06

Performance evaluated based on average percent volume overlap (PVO) and percent volume difference (PVD).

## Cell classification of ANA images

By considering the effect of astigmatism, the texture details of the cells which are not located at the central field may be lost due to optical aberration. Hence, only the cells located in the central field, accounting to 50% of the area from the image center, are used for cell classification. A total of 3830 cells extracted from 196 images are classified into 6 different patterns, i.e. diffused (599), peripheral (529), nucleolar (94), coarse-speckled (1956), fine-speckled (56), and discrete-speckled (596), by an experienced physician, Dr. Hsieh. The classified cell patterns are adopted as the ground truth to verify classification performance of the proposed method.

### Features for cell classification

For the purpose of finding suitable features to represent the patterns of ANA images, conventional and the state-of-the-art features are investigated. The conventional features used to describe patterns include statistics of intensity and texture of blobs. The statistics of blob intensity include mean, variance, skewness, and entropy. Tamura features, including coarseness, contrast, and directionality, as well as the Haralick features, including contrast, correlation, energy, and homogeneity, obtained from co-occurrence matrix (GLCM) with 0, 45, 90, and 135 degrees, are also used to characterize the blobs. Furthermore, the most frequently used state-of-the-art features, such as fuzzy texture spectrum (FTS) [27,28] and local binary pattern (LBP) [29-31], are also adopted for cell classification in this study.

**Figure 18:**
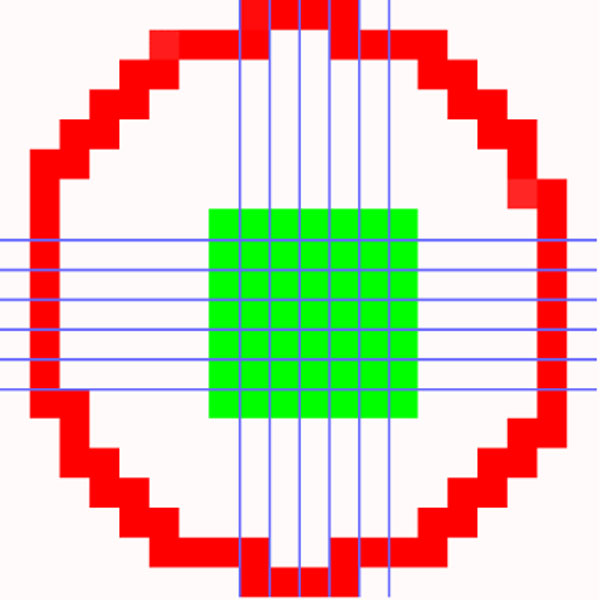
**Intensity difference between perimeter (red) and central area (green)**.

In addition, by observing the ANA images, a novel feature has been proposed to describe the appearance of blobs from the intensity images. As illustrated in Figure 18, the features obtained from the perimeters and the central areas of the blobs are different between two different cell patterns, such as the peripheral and nucleolar patterns. It can be distinguished by calculating the intensity difference between the perimeter and the central area of a blob according to the following equation:

$$d_{pc}=P_{avg}-C_{avg}$$

where *P_avg _*denotes the average intensity of pixels located at the perimeter of a blob, and *C_avg _*indicates the average intensity of the central area with a size of 7×7 pixels.

By observing images in Figure 1, it can be found that different cell patterns contain a variety of regions with different sizes and patterns. For example, although nucleolar and discrete-speckled patterns both contain light regions, the number of light regions in the cells with discrete-speckled pattern is greater than the nucleolar pattern. In contrast, some dark regions can be observed in the coarse-speckled and fine-speckled patterns. These are important and useful characteristics to reduce false cases in discriminating cells with different patterns. A total of 6 features derived from statistics of light and dark regions inside the blobs, including numbers of dark and light regions as well as mean and variance of intensity of dark and light regions, are obtained for cell discrimination.

In total, 129 candidate features were used to represent the patterns of individual ANA images. As indicated in Table 3, the features were grouped into 11 categories, i.e. STATS (3 features), TAMURA (3 features), HARALICK (16 features), FTS (45 features), LR (3 features), DR (3 features), LBP8 (10 features), LBP16 (18 features), LBP24 (26 features), ENTROPY (1 feature) and DPC (1 feature).

**Table 3:** Categories of features used for cell classification.

Category	Features No.	Description
STATS	3	Intensity statistics of blobs: mean, variation, and skewness

TAMURA	3	Coarseness, contrast, and directionality

HARALICK	16	Contrast, correlation, energy, and homogeneity in co-occurrence matrix with degrees 0, 45, 90, and 135.

FTS	45	Fuzzy texture spectrum based on the relative intensity levels among pixels

LR	3	Statistics of light regions: No. of regions as well as mean and variation of intensity

DR	3	Statistics of dark regions: No. of regions as well as mean and variation of intensity

LBP8	10	Calculated based on 8 neighbors the distance of one

LBP16	18	Calculated based on 16 neighbors the distance of one

LBP24	26	Calculated based on 24 neighbors the distance of one

ENTROPY	1	Intensity statistic of blobs

DPC	1	Intensity difference between perimeter and central area

Total	129	

## Design and validation of cell classifier

Support vector machine (SVM) is a supervised learning method widely used for classification of data patterns [32,33]. A special property of SVM is that it can simultaneously minimize the empirical classification error and maximize the geometric margin of a classifier. It is a powerful methodology for solving problems in nonlinear classification, function estimation, and density estimation, leading to many applications [34].

In this study, SVM classifier was implemented by the LIBSVM tool [35] which supports multi-class classification. Radial basis function (RBF) was selected as the kernel because of its advantages in mapping samples into a higher dimensional space so that it can handle the case when the relation between class labels and attributes is nonlinear [36]. The optimal combination of penalty parameters, *C *and *γ *of the RBF kernel, were determined by dividing the range 2^-10^-2^+10 ^into 21 steps, resulting in a total of 441 combinations.

Two experiments for the verification of classification performance of the SVM classifier were conducted: cross validation (CV) and independent training and testing (ITT). For the CV experiment, 5-fold cross-validation was conducted to obtain the optimal parameters *C *and *γ *in the training phase. On the other hand, the images dataset was randomly divided into training set and testing set, each containing 50% of the randomly selected images, for ITT. Again in the training phase, 5-fold cross-validation was used to obtain the optimal combination of parameters *C *and *γ *based on the training set. The ITT experiment were repeated for 10 times.

Table 4 reveals the resulting accuracy obtained from the CV and 10 ITTs with all of features presented in Table 3. It mimics that the proposed segmentation method is good enough to detect cell contours for extracting features to design a classifier with satisfactory classification accuracy. Additionally, one of the objectives of this study is to select salient features to represent cell patterns.

**Table 4:** Classification accuracies (%) of different cell patterns

**Exp**.	**Iter**.	All	Diffused	Peripheral	Nucleolar	Coarse-speckled	Fine-speckled	Discr.-speckled
1	-	99.69	98.33	100	98.94	99.95	100	100

2	1	97.65	93.98	99.24	82.98	98.88	85.71	99.33
	2	96.45	88.96	98.48	95.74	98.57	71.43	97.65
	3	97.23	93.65	97.72	87.23	98.47	78.57	99.66
	4	96.45	92.31	98.10	82.98	98.36	78.57	96.64
	5	97.13	94.98	96.96	89.36	98.47	71.43	98.66
	6	96.81	93.98	99.24	76.60	98.26	85.71	96.98
	7	96.81	94.31	97.34	87.23	98.67	82.14	95.64
	8	97.33	93.65	98.48	91.49	98.98	78.57	97.32
	9	96.50	90.30	98.48	93.62	98.47	85.71	95.97
	10	96.50	90.64	98.48	85.10	98.36	82.14	97.65
	
	Avg	96.87	92.68	98.25	87.23	98.55	80.00	97.55

Classified accuracies of 6 patterns of cells and overall cells achieved for CV and ITT experiments.

**Figure 19:**
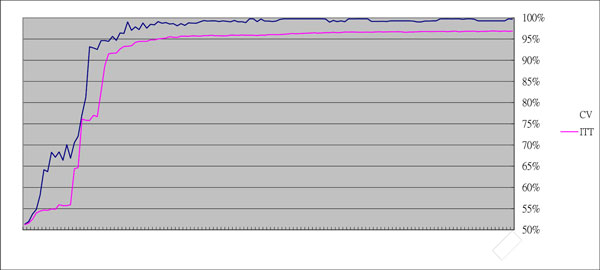
**Comparison of accuracy against different number of features**. Features selected using SVM-RFE method compared between cross validation (CV) and independent training and testing (ITT) experiments.

The sequential backward selection (SBS) [37] was frequently used in many researches for feature selection. In this study, the SVM-RFE (recursive feature elimination) reported to be effective for multi-cluster classification [38], was adopted to eliminate unimportant features according to the minimum redundancy and maximum relevancy (MRMR) criterion [39]. It was implemented with MIToolbox (Matlab version) [26]. As shown in Figure 19, the best average accuracy obtained is 99.76% with 60 features selected for designing the classifier in the CV experiment, while it achieves 96.90% accuracy for the classifier designed using 124 selected features in the ITT experiment.

## Discussion

Cytology evaluation has been shown to be a safe, efficient and well-established technique for the diagnoses of many diseases. Its ability to reduce the mortality and morbidity of cervical cancer through mass screening is the most famous success. Classical cytological diagnosis is based on microscopic observation of specialized cells and qualitative assessment with descriptive criteria, which may result in inconsistent results because of subjective variability found in different observers [40]. Recently, automatic or semi-automatic computerized systems developed for segmenting and analyzing stained cervical cells from Pap smear images are demonstrated to be effective and efficient to assist pathologists in the diagnosis of abnormal cells [34,41-43] and in the discrimination of different types of cells [34,44,45] through accurate and objective measurements of cell texture and morphology.

Tracing the cell migration, cell cycle, and cell differentiation from fluorescent microscopic images through automatic segmentation, classification, and tracking of living and cultured cells has also been widely conducted [46-48]. However, an automated image analysis system developed to fit a specific type, assay, or image set is hardly applicable to different cells acquired from different modalities [49]. Hence, techniques used for segmenting cells from visible-light microscopic images may not be directly applied in extracting cells from fluorescent microscopic images, whereas techniques used for extracting cells in a living cell population from fluorescent microscopic images may not be effective for processing IIF images.

Tested with the 3830 cells extracted from 196 images, the segmentation results show that PVO is greater than 89% and PVD is less than 22%. The average classification accuracy achieved in this study is as high as 96.90% (error rate: 3.1%) and 99.69% (error rate: 0.31%) for CV and ITT experiments, respectively, which outperforms the performance reported in previous studies [3,5,6,16-19]. Table 5 compares the cell/image numbers and the error rates in classification of this study with other previous investigations.

**Table 5:** Comparison of error rate between this study and previous investigations.

Literature	Error rate	No. of cells/images	Validation method
Perner et al. [6]	23.60%	NA/321	Human expert
	25.00%	NA/321	leave-one-out CV
Sack et al. [3]	16.91%	1041/676	CV
Elbischger et al. [17]	9.75%	982/38	ITT (1:1)
Soda & Iannello [5]	5.80%	573/37	8-fold CV
Rigon et al. [16]	26.00%	573/37	8-fold CV
Huang et al. [18]	7.60%	1020/NA	10-fold CV
Voigtet al. [19]	6.00%	NA/351	ITT
This study	0.31%	3830/196	5-fold CV
	3.10%	3830/196	ITT (1:1)

CV indicates cross validation and ITT denotes independent training and testing.

**Figure 20:**
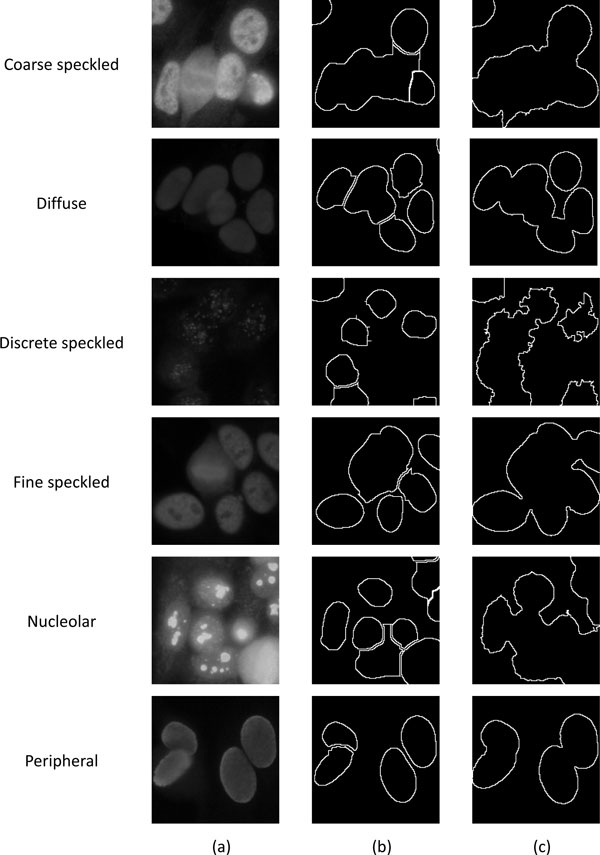
**Comparison of segmentation outcome between proposed method and CellProfiler**. (a) Original images of 6 different ANA patterns and their segmented results using (b) proposed method and (c) CellProfiler.

Note that the cells included in the database used in the this study are quite different from the cells adopted in previous studies, which may induce bias when making comparisons. CellProfiler is a freely available software [49] useful for automatic cell segmentation as well as for quick and easy classification and scoring of cells with diverse cellular morphologies [48]. Figure 20 compares the segmented cell examples between the proposed method and the CellProfiler. It can be observed that the proposed method outperforms CellProfiler regarding the individual cells categorized into 6 different patterns.

In addition to PVO and PVD, other evaluation criteria, including, relative foreground area error (RAE) [50] and modified Hausdorff distance (MHD) [51], are also used to measure the segmentation errors. As can be seen in Table 6, the proposed method demonstrates better segmentation performance over the CellProfiler when evaluated based on PVO, PVD, RAE, and MHD. In addition, the number of miss-segment cells of the proposed method is less than the CellProfiler.

**Table 6:** Comparisons of number of segmented cells and classification performance between proposed method and CellProfiler evaluated based on PVO, PVD, RAE, and MHD.

Pattern	Detected Cell Number			PVO (%)		PVD (%)		**RAE ± Std**.		**MHD ± Std**.	
	
	G. Truth	Proposed	CellProfiler	Proposed	CellProfiler	Proposed	CellProfiler	Proposed	CellProfiler	Proposed	CellProfiler
Diffuse	251	157	107	87.89	84.75	24.23	30.50	0.6386 ± 0.42	0.5248 ± 0.36	250.90 ± 228.42	510.69 ± 250.56
Peripheral	191	153	97	93.47	72.43	13.06	55.15	0.3039 ± 0.35	0.5120 ± 0.31	87.15 ± 182.86	602.36 ± 267.78
Nucleolar	285	175	101	91.94	84.90	16.12	30.20	0.4777 ± 0.41	0.6445 ± 0.33	157.99 ± 208.70	560.73 ± 225.77
Coarse speckled	363	260	150	92.35	87.84	15.31	24.32	0.3506 ± 0.36	0.7038 ± 0.21	101.90 ± 199.74	556.02 ± 261.51
Fine speckled	117	67	36	91.27	91.22	17.46	17.55	0.5423 ± 0.41	0.8637 ± 0.13	173.20 ± 13.16	458.46 ± 21.41
Discrete speckled	216	195	125	78.03	69.64	43.95	60.71	0.5457 ± 0.37	0.7277 ± 0.18	143.99 ± 202.59	504.55 ± 242.68

## Conclusion and future work

In this study, a segmentation method was proposed to detect the boundaries of HEp-2 cells automatically, and then classification of cell patterns was performed based on the selected features. The results show that the proposed method can detect cells correctly in most image cases with PVO greater than 89% and PVD less than 22%, whereas the best combination of selected features can achieve an average accuracy as high as 96.90% in discriminating 6 different types of cell patterns.

More cell images will be included in the dataset for verifying the segmentation performance and classification performance in the future. Furthermore, an automatic segmentation and classification system with graphical user interface (GUI) will be developed for computer-aid diagnosis. In fact, several different ANA patterns can appear in a single image, but the segmentation method proposed here only considers images with a unique cell pattern. Future works will focus on developing a segmentation method to extract cells with different patterns appearing in an image.

## Competing interests

The authors declare that they have no competing interests.

## Authors' contributions

CCC designed the software and conducted the image analysis; TYH recruited the patients, acquired the images, and verified the experimental results; CCC, JST, and YFC contributed to the discussion of the work and wrote the paper. All authors read and approved the final manuscript.
